# Supplementary material for: An anionic phthalocyanine decreases NRAS expression by breaking down its RNA G-quadruplex
Source: Nat Commun. 2018 Jun 11;9:2271. doi: 10.1038/s41467-018-04771-y (PMC5995912; doi:10.1038/s41467-018-04771-y)
Supplement: Supplementary file 1 — Supplementary Information [file 41467_2018_4771_MOESM1_ESM.pdf]

An anionic phthalocyanine decreases NRAS expression by breaking down its RNA G-quadruplex

Kawauchi et al.

Supplementary Information contains:

Supplementary Figures 1-10

Supplementary Table 1

Supplementary Figures

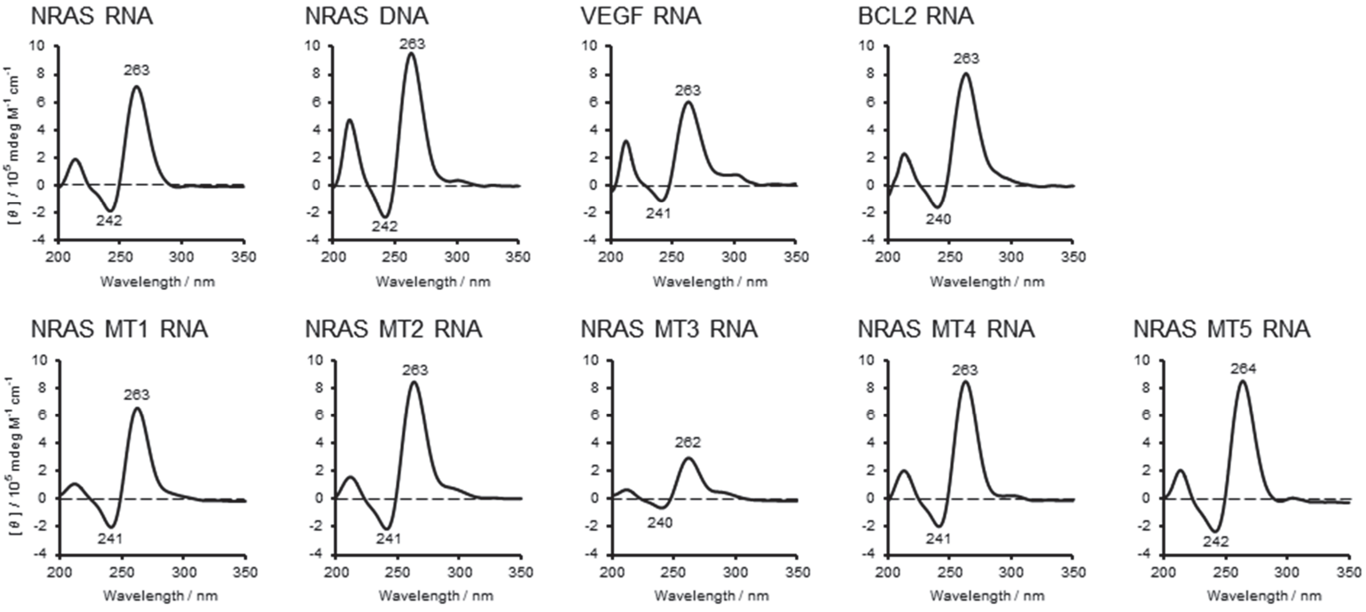

Supplementary Figure 1. CD spectra of 20 μM RNA and DNA G-quadruplexes used in this study.

CD spectra of 20 μM NRAS RNA, NRAS DNA, VEGF RNA, BCL2 RNA, NRAS MT1 RNA, NRAS MT2 RNA, NRAS MT3 RNA, NRAS MT4 RNA, and NRAS MT5 RNA at 25°C. All the CD spectra show positive and negative peaks around 260 nm and 240 nm, respectively, which are a signature of a parallel G-quadruplex.

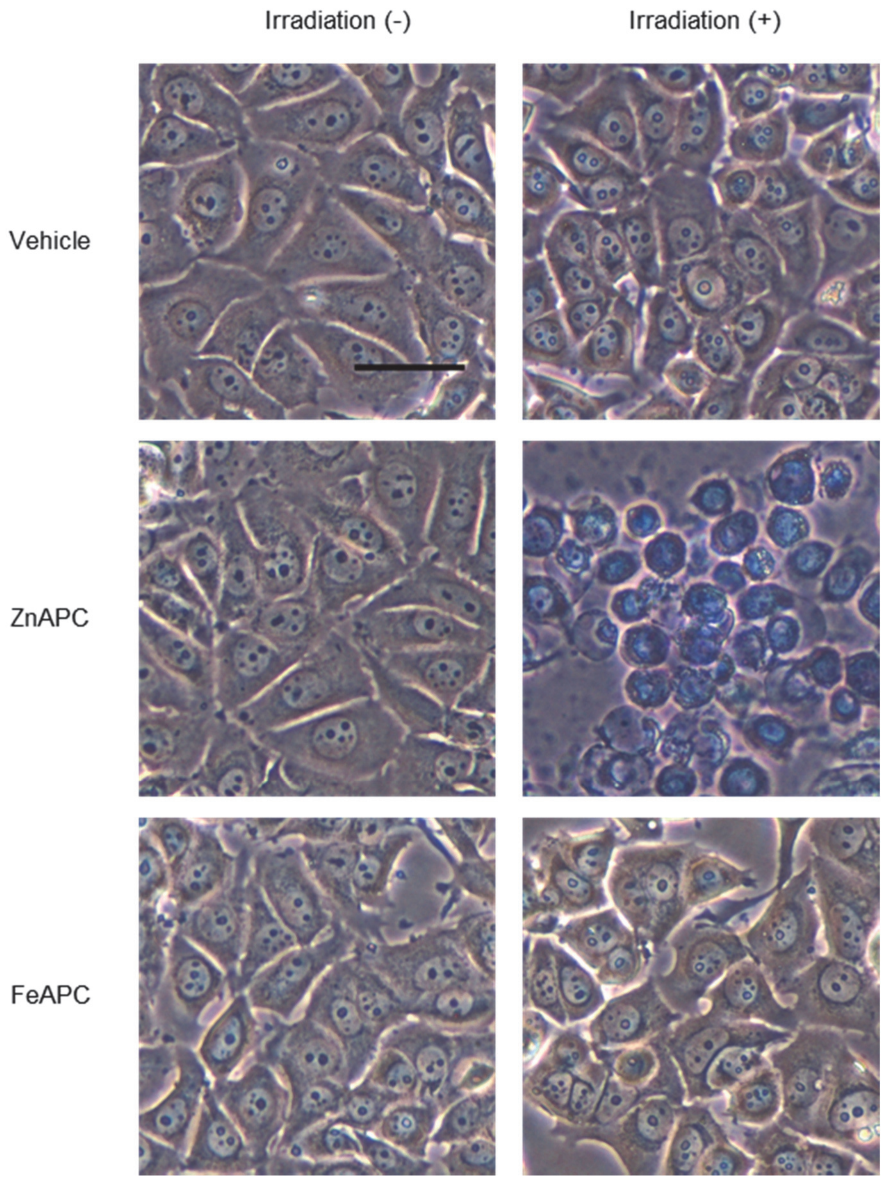

Supplementary Figure 2. ZnAPC induces cell death upon photo-irradiation.

Cells pre-treated with 10 μM ZnAPC or FeAPC for 1 h were incubated for 24 h after photo-irradiation for 2 h. Phase contrast images of cells stained with Trypan Blue to identify dead cells are presented. Scale bar, 50 μm.

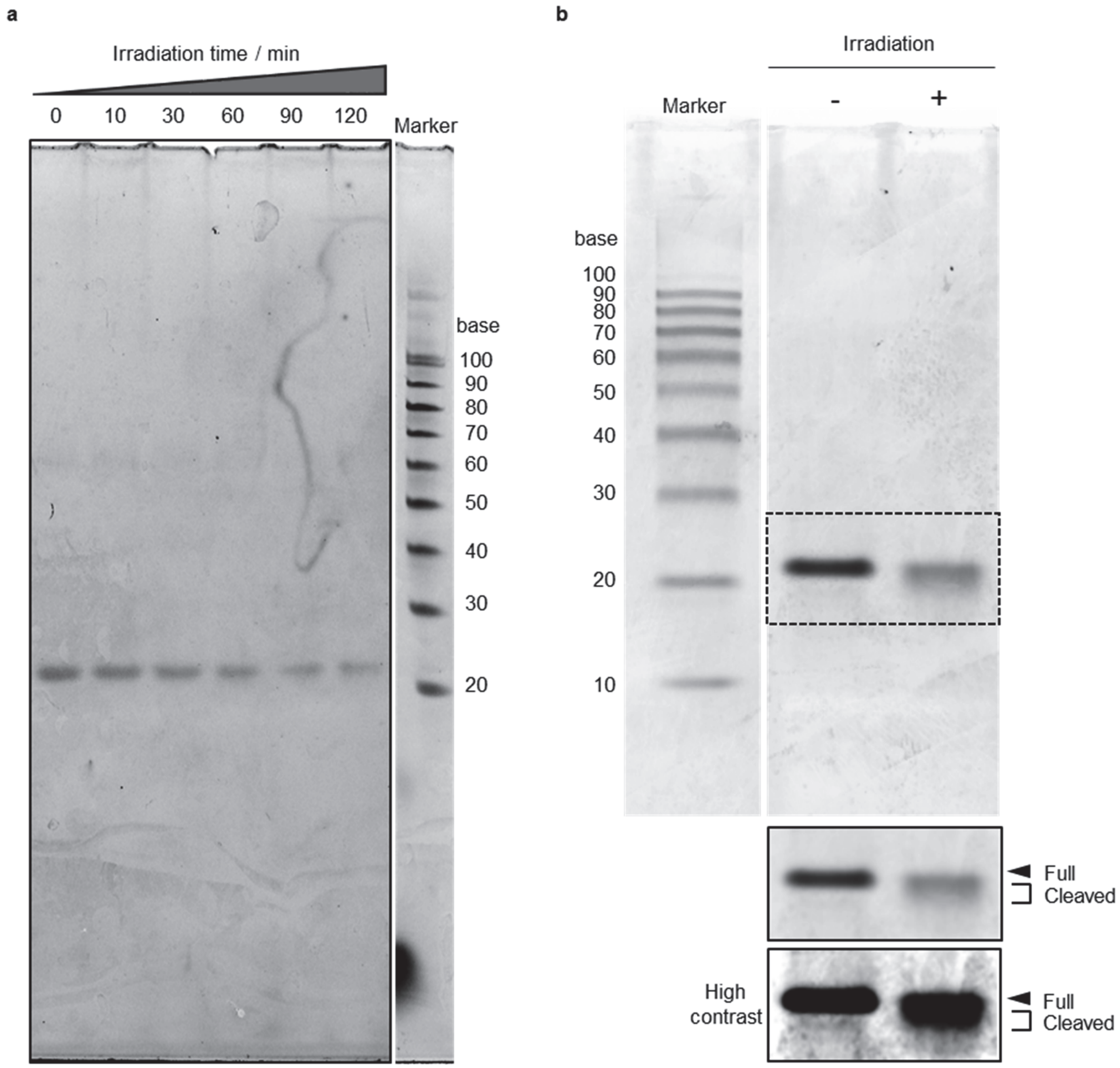

Supplementary Figure 3. ZnAPC cleaves NRAS RNA upon photo-irradiation.

a The full image of 10% denaturing polyacrylamide gel that is shown in Figure 3a for NRAS RNA. **b** Electrophoresis (in a 15% denaturing polyacrylamide gel) of 0.1 μM NRAS RNA in the presence of 2 μM ZnAPC after photo-irradiation for 120 min. The loading amount was two times that shown in Figure 3a. The middle or bottom panels are expanded images of a portion of the full gel (top panel), which is enclosed in a dotted rectangle.

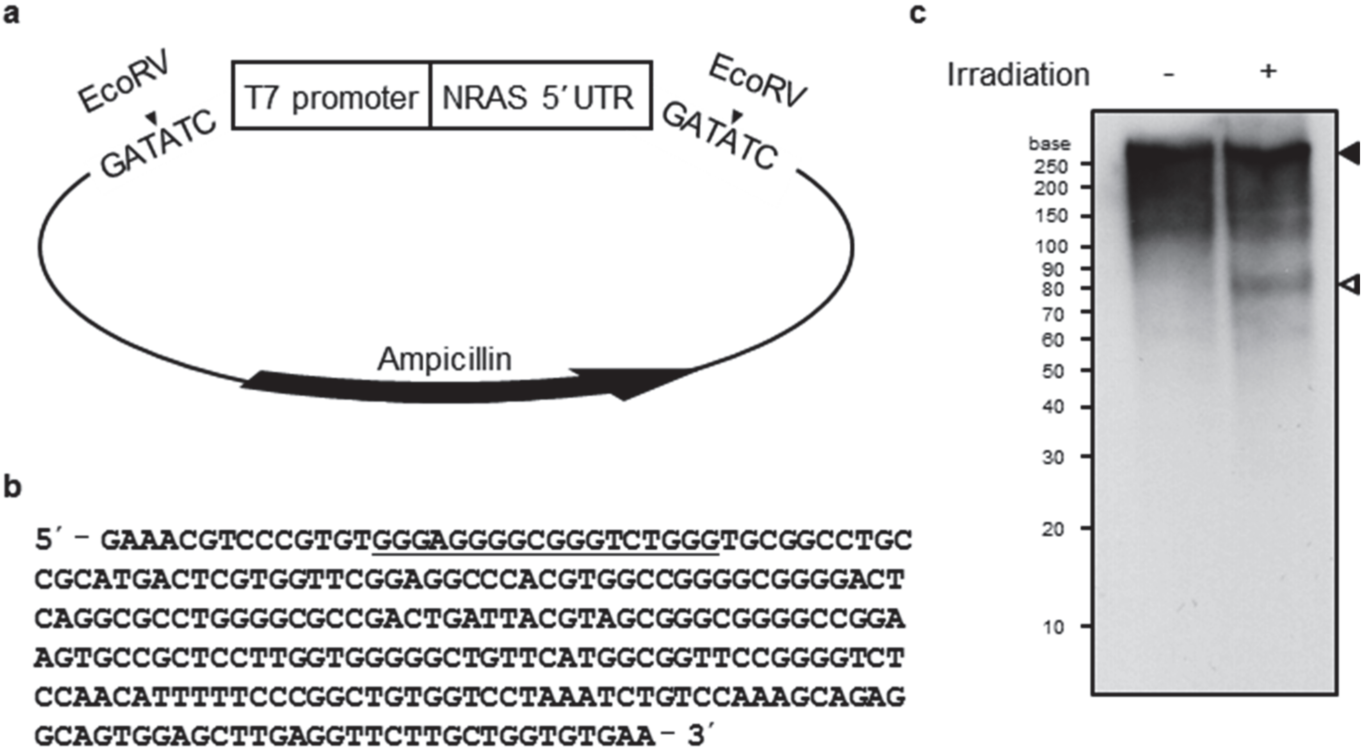

Supplementary Figure 4. ZnAPC cleaves NRAS F-RNA upon photo-irradiation.

a The plasmid map of the pUC57 NRAS F-RNA vector containing sequence encoding the 5′ UTR of *NRAS* mRNA following the T7-promoter. **b** The sequence of 5′ UTR in *NRAS* mRNA. The underlining indicates a G-quadruplex–forming sequence. **c** A long-exposure full image of the membrane shown in Figure 3c for NRAS F-RNA. Black and white arrow heads indicate the full-length and cleaved fragment of NRAS F-RNA, respectively.

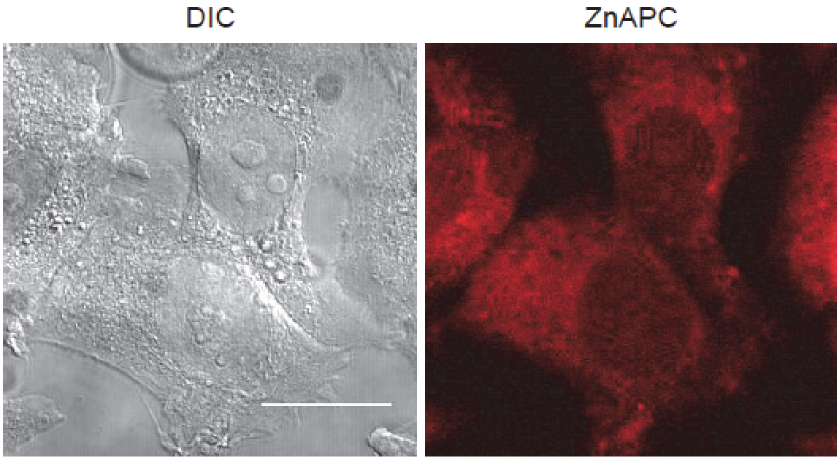

Supplementary Figure 5. ZnAPC diffuses throughout the cytosol in the cells.

Distribution of ZnAPC in a cell was evaluated by its autofluorescence. Cells were treated with 10 μM ZnAPC for 3 h. DIC image and fluorescence image of ZnAPC are shown. Scale bar, 20 μm.

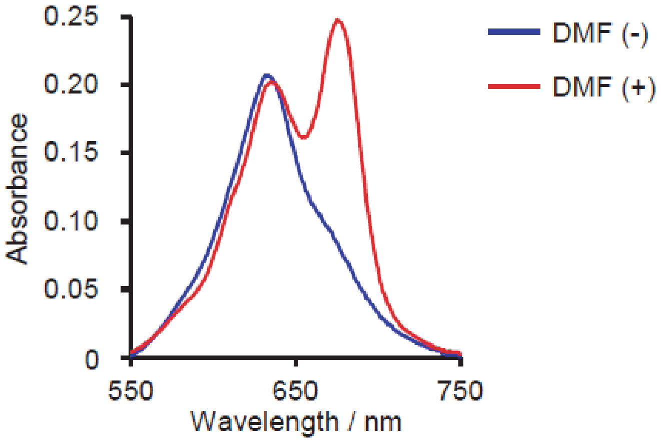

Supplementary Figure 6. VIS absorbance spectra of ZnAPC with DMF.

VIS absorbance spectra of 2 μM ZnAPC in the buffer consisting of 50 mM MES-LiOH (pH 7.0) and 100 mM KCl at 25 °C in the absence or presence of 30 wt% DMF. The addition of 30 wt% DMF monomerises ZnAPC.

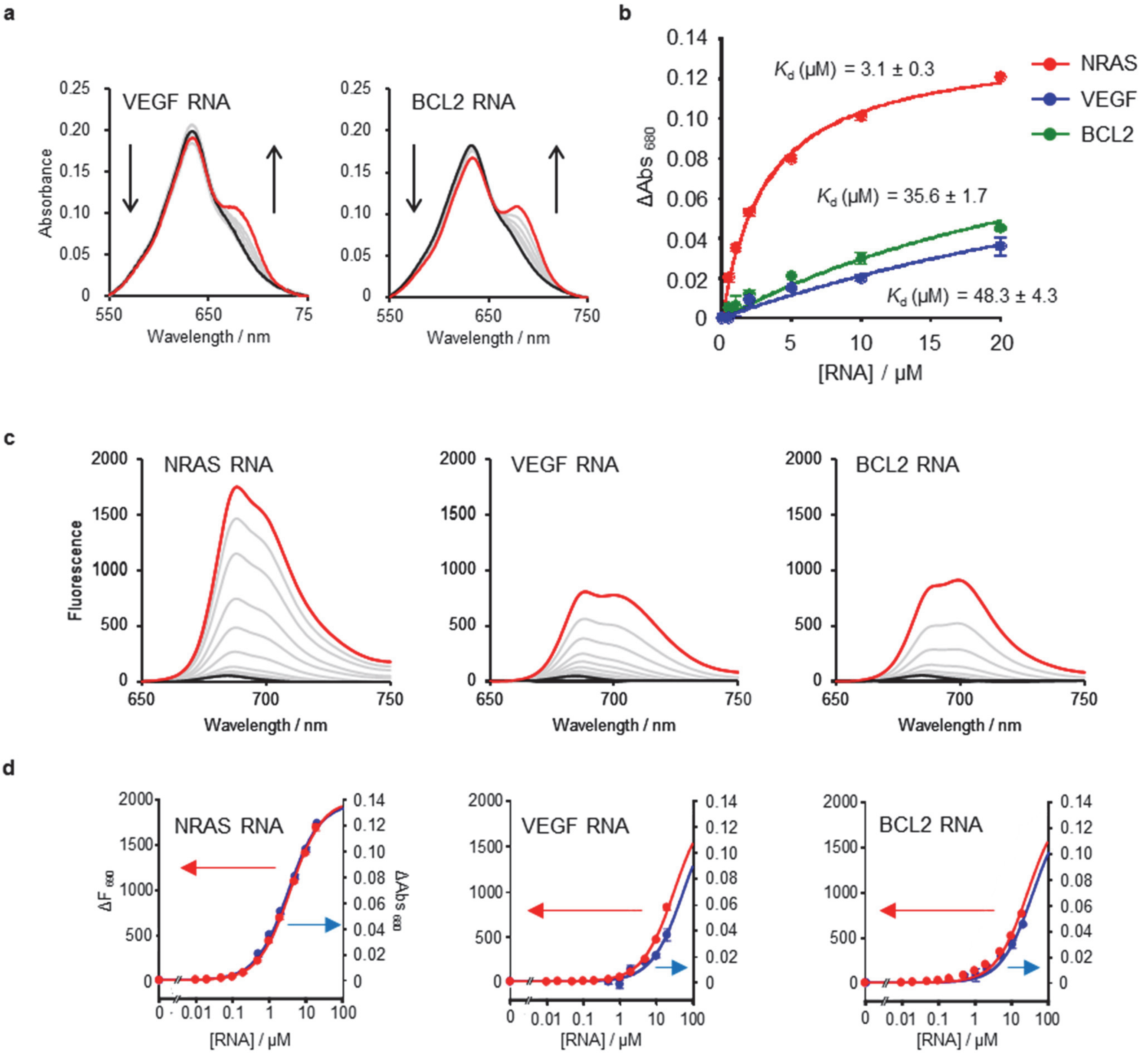

e

	NRAS RNA	VEGF RNA	BCL2 RNA
$K_{d, Abs}$ (μM)	3.1 ± 0.3	48.3 ± 4.3	35.6 ± 1.7
$K_{d, F}$ (μM)	4.0 ± 0.1	31.2 ± 1.2	28.6 ± 0.5

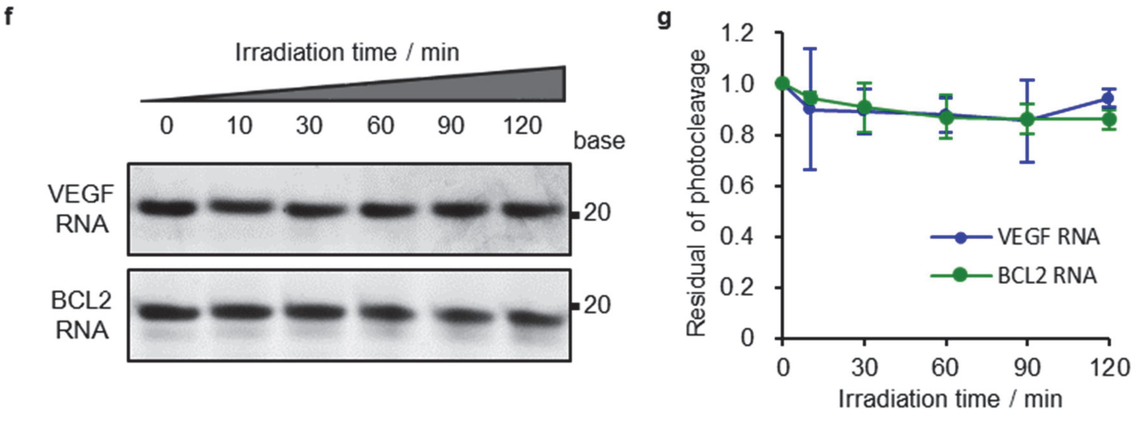

Supplementary Figure 7. ZnAPC does not cleave VEGF RNA and BCL2 RNA.

a VIS absorbance spectra of 2 µM ZnAPC with 0, 0.5, 1, 2, 5, 10, or 20 µM VEGF RNA or BCL2 RNA at 25 °C. The spectra for 0 and 20 µM RNA are highlighted in black and red, respectively. **b** Plots of Δabsorbance at 680 nm (= absorbance with NRAS RNA minus absorbance without NRAS RNA) of ZnAPC in comparison with the concentration of NRAS RNA (red), VEGF RNA (blue), or BCL2 RNA (green). NRAS RNA data were duplicated from Fig. 1b for comparison. Continuous curves are the results of curve-fitting to a theoretical equation, and the estimated K_d values are also shown. **c** Fluorescence spectra of 2 µM ZnAPC with 0, 0.01, 0.02, 0.05, 0.1, 0.2, 0.5, 1, 2, 5, 10, or 20 µM NRAS RNA, VEGF RNA, or BCL2 RNA at 25 °C. The spectra with 0 and 20 µM are highlighted in black and red, respectively. **d** Plots of Δfluorescence intensity at 690 nm (= fluorescence intensity with RNA minus fluorescence intensity without RNA) of ZnAPC vs. the concentration of NRAS RNA, VEGF RNA, or BCL2 RNA. Data on Δabsorbance at 680 nm shown as the Y2 axis are also illustrated to compare these data with the changes in the Δfluorescence intensity at 690 nm. Continuous curves denote results of curve-fitting to a theoretical equation. **e** K_d values of ZnAPC with the RNAs evaluated from the titration results traced by Δabsorbance at 680 nm ($K_{d \cdot Abs}$) and Δfluorescence intensity at 590 nm ($K_{d \cdot F}$). **f** Electrophoresis (in a 10% denaturing polyacrylamide gel) of 0.1 µM VEGF RNA and BCL2 RNA in the presence of 2 µM ZnAPC after photo-irradiation for the indicated periods. **g** Residual intact RNA after the photo-cleavage of VEGF RNA and BCL2 RNA. Error bars represent mean ± SD; n = 3.

a

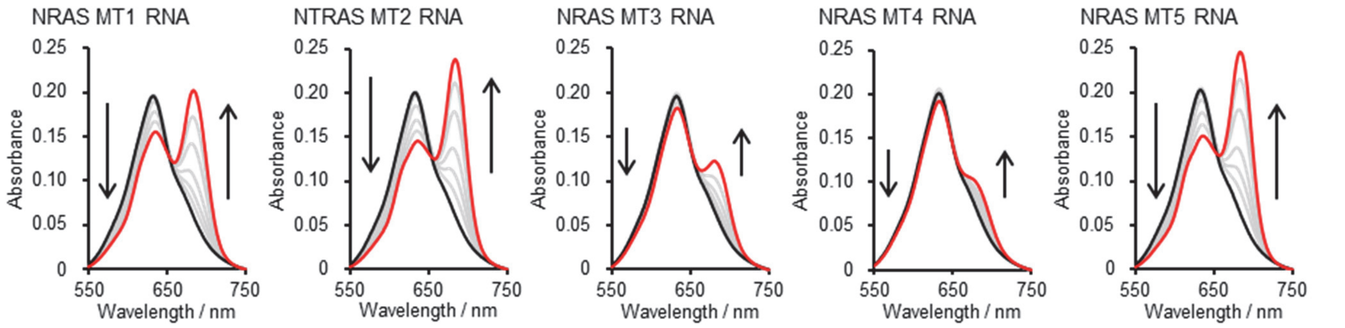

b

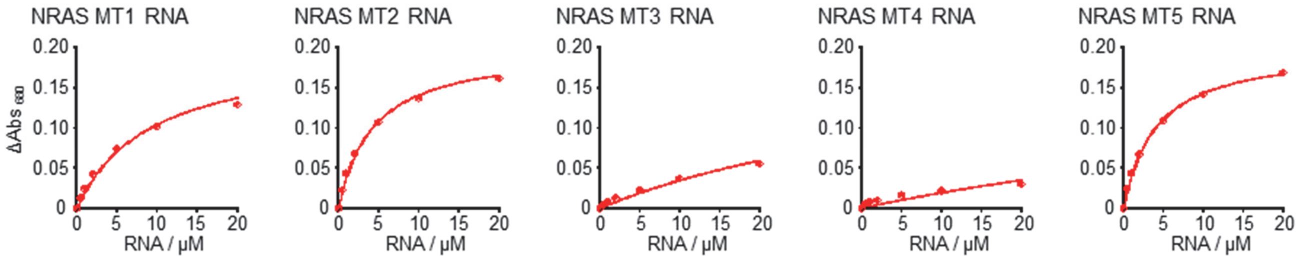

c

RNA	NRAS MT1	NRAS MT2	NRAS MT3	NRAS MT4	NRAS MT5
K_d (μM)	9.2 ± 0.1	4.2 ± 0.2	48.0 ± 1.0	95.4 ± 5.0	4.0 ± 0.02

Supplementary Figure 8. The binding affinity of ZnAPC for NRAS RNA mutants.

a VIS absorbance spectra of 2 μM ZnAPC with 0, 0.5, 1, 2, 5, 10, or 20 μM NRAS MT1 RNA, NRAS MT2 RNA, NRAS MT3 RNA, NRAS MT4 RNA, or NRAS MT5 RNA at 25 °C. The spectra with 0 and 20 μM RNA are highlighted in black and red, respectively. **b** Plots of Δabsorbance at 680 nm (= absorbance with NRAS RNA minus absorbance without NRAS RNA) of ZnAPC vs. the concentration of the RNAs. Error bars represent mean ± SD; n = 3. Continuous curves are results of curve-fitting to a theoretical equation to evaluate the K_d values of ZnAPC with the RNAs. The calculated values in panel **b** at 25 °C are listed in panel **c**.

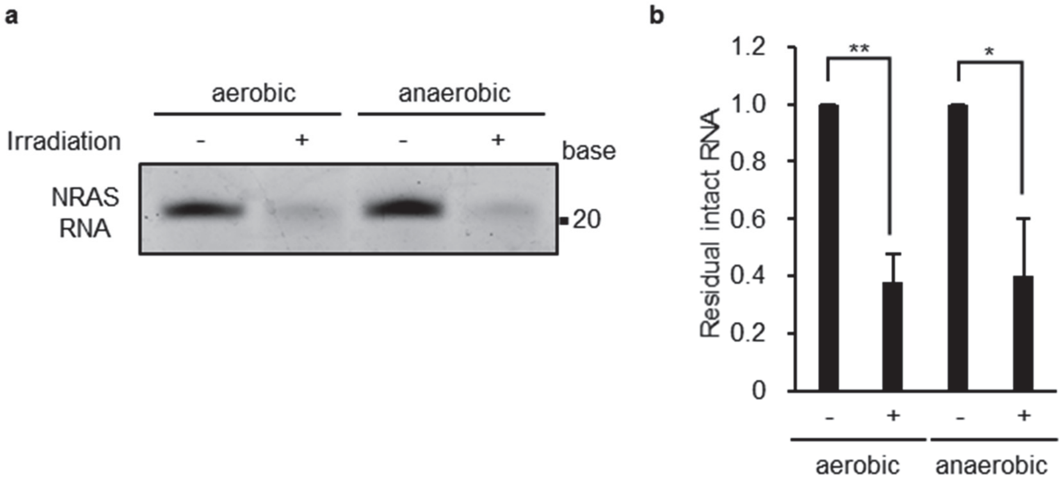

Supplementary Figure 9. ZnAPC cleaves NRAS RNA under both aerobic and anaerobic conditions upon photo-irradiation.

a Denaturing polyacrylamide gel electrophoresis of 0.1 μM NRAS RNA in a 10% gel in the presence of 2 μM ZnAPC with or without photo-irradiation for 2 h under aerobic or anaerobic conditions. **b** Residual intact RNA after the photo-cleavage of NRAS RNA. Each bar represents mean ± SD; n = 3. For statistical significance, an unpaired t-test was performed. *$p < 0.01$; **$p < 0.001$.

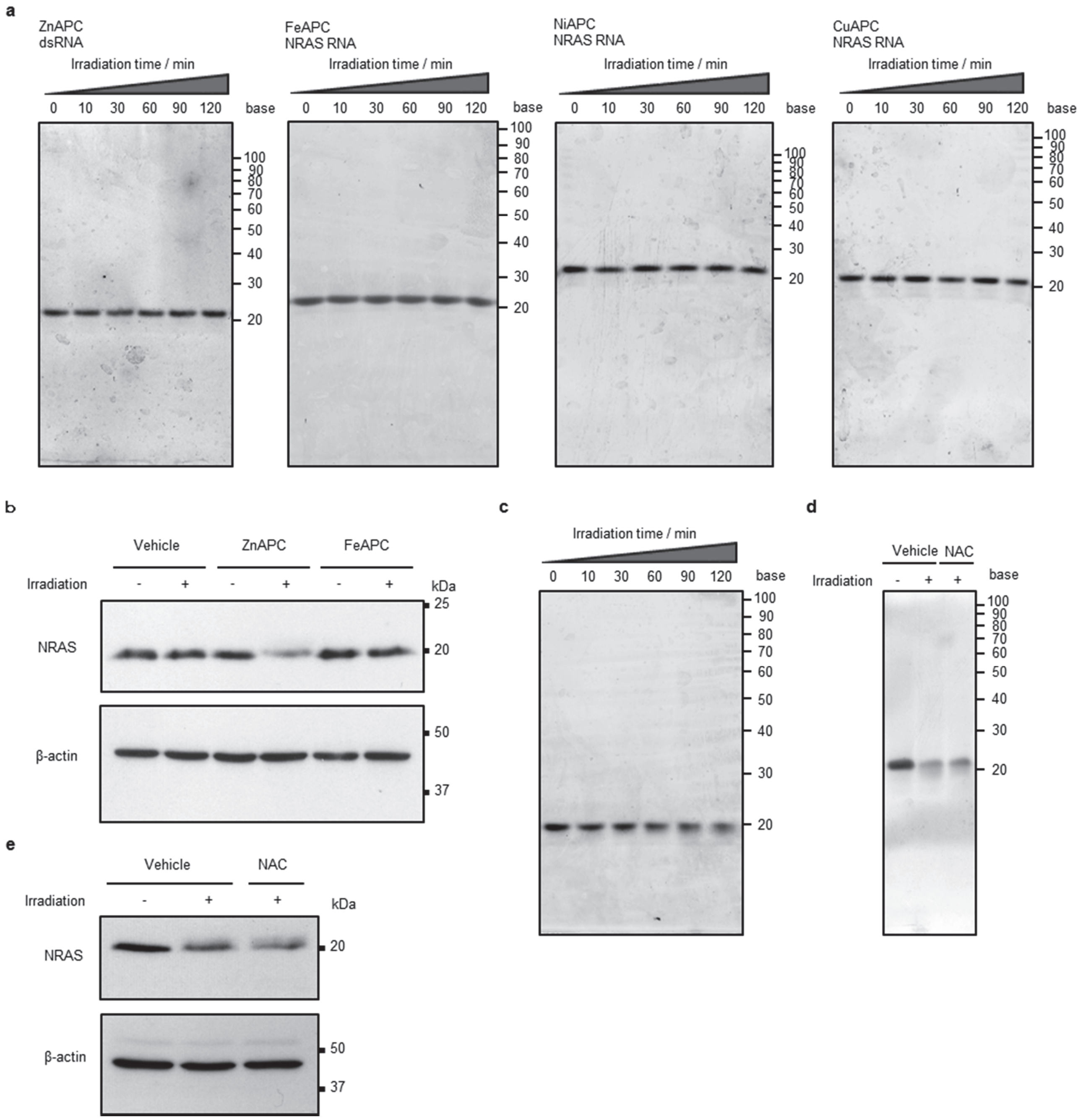

Supplementary Figure 10. Uncropped immunoblot and gel images.

a Figure 3a, **b** Figure 3e, **c** Figure 4b, **d** Figure 5d, **e** Figure 5g.

Supplementary Table

Supplementary Table 1. Sequences of oligonucleotides used in this study.

	Sequence	Secondary structure
NRAS RNA	GGGAGGGGCGGGUCUGGG	Parallel G-quadruplex
dsRNA	AGUUCAAGGCGCCUUGAACU	Duplex
NRAS DNA	GGGAGGGGCGGGTCTGGG	Parallel G-quadruplex
VEGF RNA	GGAGGAGGGGAGGAGGA	Parallel G-quadruplex
BCL2 RNA	GGGGGCGGUGGGGUGGGAGCUGGGG	Parallel G-quadruplex
NRAS MT1 RNA	GGGAGGAGGGGGAGGGAGGG	Parallel G-quadruplex
NRAS MT2 RNA	GGGCGGUGGGGUGGGAGCUGGG	Parallel G-quadruplex
NRAS MT3 RNA	GGAGGGCGGUCUGG	Parallel G-quadruplex
NRAS MT4 RNA	GGGGAGGGGCGGGUCUGGGG	Parallel G-quadruplex
NRAS MT5 RNA	UGGGAGGGGCGGGUCUGGGU	Parallel G-quadruplex
